# Development of a Real-Time Recombinase-Aided Amplification Method to Rapidly Detect Methicillin-Resistant *Staphylococcus aureus*

**DOI:** 10.3390/microorganisms10122351

**Published:** 2022-11-28

**Authors:** Xiaoyan Ding, Hejia Wang, Mingquan Cui, Min Cheng, Qi Zhao, Yuhui Bai, Chunping Zhang, Cunshuai Zhang, Shixin Xu, Ting Li

**Affiliations:** Key Laboratory of Animal Antimicrobial Resistance Surveillance, Ministry of Agriculture and Rural Affairs, National Reference Laboratory of Veterinary Drugs Residues, China Institute of Veterinary Drug Control, Beijing 100081, China

**Keywords:** MRSA, a real-time RAA, rapid detection, qPCR

## Abstract

Methicillin-resistant *staphylococcus aureus* (MRSA) is a major pathogen responsible for human hospital and community-onset diseases and severe invasive livestock infections. Rapid detection of MRSA is essential to control the spread of MRSA. Conventional identification methods and antibacterial susceptibility tests of MRSA are time-consuming. The commonly used qPCR assay also has the disadvantages of being complicated and expensive, restricting its application in resource-limited clinical laboratories. Here, a real-time fluorescent recombinase-assisted amplification (RAA) assay targeting the most conserved regions within the *mecA* gene of MRSA was developed and evaluated to detect MRSA. The detection limit of this assay was determined to be 10 copies/reaction of positive plasmids. The established RAA assay showed high specificity for MRSA detection without cross-reactivities with other clinically relevant bacteria. The diagnostic performance of real-time RAA was evaluated using 67 clinical *S. aureus* isolates from dairy farms, which were detected in parallel using the TaqMan probe qPCR assay. The results showed that 56 and 54 samples tested positive for MRSA by RAA and qPCR, respectively. The overall agreement between both assays was 97.01% (65/67), with a kappa value of 0.9517 (*p* < 0.001). Further linear regression analysis demonstrated that the detection results between the two assays were significantly correlated (*R*^2^ = 0.9012, *p* < 0.0001), indicating that this RAA assay possesses similar detection performance to the qPCR assay. In conclusion, our newly established RAA assay is a time-saving and convenient diagnostic tool suitable for MRSA detection and screening.

## 1. Introduction

*S. aureus* is a major pathogenic bacteria widely distributed in nature that mainly causes superficial skin and soft tissue infections, surgical wood infections, and sometimes-fatal bloodstream infections and pneumonia [1,2]. Since the first discovery of methicillin-resistant *S. aureus* (MRSA) in the United Kingdom in 1961 [3], MRSA has been widely distributed worldwide. Resistance of MRSA to β-lactam antibiotics is mediated by PBP2a, a penicillin-binding protein with low affinity to β-lactams, encoded by the *mecA* gene located at the staphylococcal cassette chromosome *mec* (*SCCmec*) [4,5,6,7,8]. In humans, MRSA is a major pathogen responsible for hospital outbreaks (health-care-associated MRSA (HA-MRSA)) and community-onset disease (community-associated MRSA (CA-MRSA)) [9,10,11]. Apart from humans, livestock-associated MRSA (LA-MRSA) not only widely exists in livestock and poultry, pets, and wild terrestrial and aquatic animals (livestock-associated MRSA (LA-MRSA)) [12,13], but it can also be transmitted through environment-human, livestock, and poultry products–human, and human–human transmission, increasing the probability of human infection [14,15]. The continuous growth in LA-MRSA infections has seriously threatened world public health security [16,17].

Rapid and accurate detection is critical for timely health care implementation and essential to limit the spread of MRSA. Conventional identification methods and drug susceptibility tests such as the disk diffusion method (KB method), broth/agar dilution method, concentration gradient method (E-test), and latex agglutination assay are all based on culture and phenotype analysis, which is time-consuming (48–72 h). They cannot meet the need for rapid detection of MRSA [17,18]. The chromogenic medium method can isolate and cultivate MRSA strains within 20–26 h, but its sensitivity may need to be further improved [19,20,21,22]. Similarly, automatic instruments have the advantage of faster detection speed but at a higher cost, and their sensitivity and specificity need to be improved when detecting low-level drug-resistant MRSA strains [23]. Real-time PCR targeting the *mecA* gene is currently the most used method for MRSA detection and screening due to its fast detection speed and high specificity [22,24,25,26,27,28,29,30]. However, it also has drawbacks, such as being complex, expensive, and requiring experienced technicians, which makes it unsuitable for extensive use in resource-limited clinical laboratories or livestock farms. Therefore, there is a need to develop a faster, more straightforward, and more user-friendly method for on-site detection of MRSA.

As a novel nucleic acid detection method, isothermal amplification technology is widely used in pathogen diagnosis due to its simplicity and rapidity [31]. It also plays an increasingly important role in detecting antimicrobial-resistant bacteria. For example, isothermal loop-mediated amplification (LAMP) has been applied in detecting extended-spectrum beta-lactamase (ESBL)-producing Enterobacteriaceae, carbapenem-resistant *A. baumannii*, macrolide–streptogramin-resistant *S. aureus* and *pcsB*-positive *S. agalactiae* [32,33,34,35,36,37]. In addition, recombinase polymerase amplification (RPA) assay has been used to detect the polymyxin-resistant gene *mcr-1*, ESBL-producing *E. coli*, *bla_NDM_-1*-positive *K. pneumoniae*, and multidrug-resistant tuberculosis [38,39,40,41,42].

Recombinase-aided amplification (RAA) assay, as an emerging isothermal amplification technology, does not require a particular thermocycling device and can be completed within 30 min at a constant low temperature (37–42 °C) [43]. The optimal RAA assay mainly depends on three crucial enzymes: a recombinase (pair-specific primers to template DNA), a strand-replacement DNA polymerase (for amplification and extension), and a single-stranded DNA-binding protein [44,45,46]. Furthermore, the amplification product of RAA can be monitored by a fluorescent probe, gel electrophoresis, lateral flow chromatography, and portable blue light instrument. In addition to being widely used in the detection of pathogenic microorganisms in humans and animals [43,45,46,47,48,49,50,51,52], RAA has also been gradually applied in the rapid detection of antimicrobial resistance genes, such as the carbapenemase gene (*bla_KPC_*) and *NDM* gene [53,54].

This study developed a fluorescent probe-based real-time RAA assay targeting a highly conserved region within the *mecA* gene to detect MRSA rapidly. Furthermore, we also compared its detection performance against a published qPCR assay using *S. aureus* isolates.

## 2. Materials and Methods

### 2.1. Bacterial Strains and DNA Extraction

Methicillin-resistant *S. aureus* (MRSA) (ATCC43300), methicillin-sensitive *S. aureus* (MSSA) (ATCC29213), *E. faecalis* (ATCC29212), *S. agalactiae* (ATCC13813), *E coli* (ATCC25922), *C. jejuni* (ATCC33560), and *S. enteritidis* (clinical isolate) were preserved in the Safety Evaluation Office of the China Institute of Veterinary Drug Control. A total of 67 *S. aureus* isolates from dairy farms were isolated from the HeNan, HeBei, and LiaoNing provinces in China (2019~2020) and identified as *S. aureus* by a *nuc* gene-specific PCR assay described in a previous report [55]. The different genomic DNAs of the cultured bacteria were extracted by boiling method. Briefly, the bacterial suspension was boiled for 10 min, centrifuged at 12,000× *g* at 4 °C for 5 min, and the supernatant was stored at −20 °C as an amplification template.

### 2.2. Plasmid

The recombinant plasmid pUC57-*mecA* was constructed by cloning the full-length *mecA* gene of the N315 strain (GenBank No. NG_047938.1) into the multiple cloning site of pUC57 using standard procedures. Plasmid pUC57-*mecA* was extracted and purified using an EndoFree Maxi Plasmid Kit (TianGen Biotech Co., Ltd., Beijing, China) according to the manufacturer’s instructions. The purified plasmid was quantified using a NanoDrop OneC Spectrophotometer (ThermoFisher, Waltham, MA, USA).

### 2.3. RAA Primer and Exo Probe

**Figure 1:**
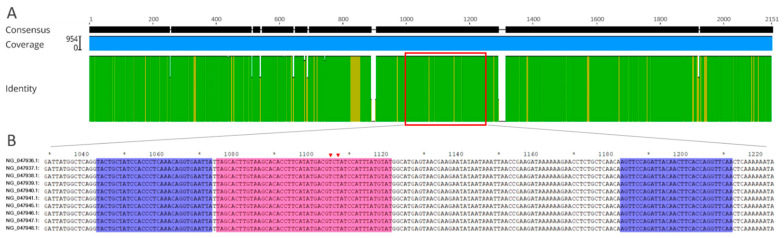
Design of the *mecA* real-time RAA primers and probe. (**A**) Schematic diagram showing the conserved region of the *mecA* gene by a multiple sequence alignment. The full-length *mecA* genes of 954 strains of MRSA were aligned using the Geneious Prime software; a conserved region (p. 1041–1210) was selected as the final target for the RAA assay is marked with a red box. (**B**) Positions of the *mecA* real-time RAA primers and probe. The specific positions of the RAA probes and primers are shown on the *mecA* gene sequences of 10 representative MRSA strains with the GenBank accession numbers on the left side. The forward primer (F1041–1071), reverse primer (R1181–1210), and exo probe (p1073–1120) are marked with blue, blue, and pink shading, respectively. The two conserved thymine (T1103, T1105) residues labeled with dT-fluorophore residue (FAM-dT) and dT-quencher residue (BHQ1-dT) within the probe are marked with solid pink triangles, respectively.

**Figure 2:**
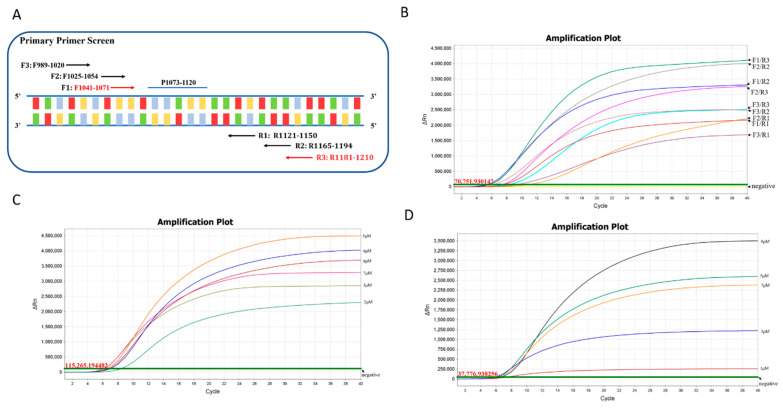
Reaction optimization for real-time RAA assays. (**A**) Schematic diagram of the primary primer screening. The numbers in the primer name represent the position of the oligonucleotides in the *mecA* gene of the N315 strain. (**B**) Representative amplification outcomes of real-time RAA in the primer pairs screening. All nine candidate primer pairs (F1/R3–F3/R3) were screened with the fixed amplification parameters. Three forward and three reverse candidate primers surrounding the probe p1073–1120 are indicated. (**C**) Optimization of the primer pair concentration for the real-time RAA assay. The amplification curves of different colors correspond to the concentrations of the primer pairs stock solution added in the reaction, including 2 µM, 3 µM, 4 µM, 5 µM, 6 µM, and 7 µM, respectively. (**D**) Optimization of the probe concentration for the real-time RAA assay. The amplification curves of different colors correspond to the concentration of the RAA probe stock solution added in the reaction, including 1 µM, 3 µM, 5 µM, 7 µM, and 9 µM, respectively.

To identify the highly conserved regions within *mecA*, 954 *mecA* sequences of *S. aureus* currently available in the NCBI database were aligned using the MAFFT Alignment program of the Geneious Prime software (Biomatters Ltd., Auckland, New Zealand). The conserved region of about 300 bp (980~1280 nt) within *mecA* was selected as the target candidate for real-time RAA assay (Figure 1A). According to the criteria suggested in the amplification guidelines (Amp-Future Biotech Co., Ltd., Weifang, Shandong, China), generally, there is a restriction of probe locations to sequences in which two thymines can be found with fewer than about five intervening nucleotides, and an optimal fragment (p1073–1120) was finally designed as the exo probe. The two conserved thymines (T1103, T1105) within the probe (p1073–1120) were labeled with dT-fluorophore residue (FAM-dT) and dT-quencher residue (BHQ1-dT), respectively (Figure 1B). After that, a series of forward and reverse candidate primers surrounding p1073–1120 were designed to proceed with a primer screening assay using the Primer Premier 5.0 software (Premier Biosoft International, Polo Alto, CA, USA) (Figure 2A). In addition, the primer pair (*mecA*-F/*mecA*-R) and the corresponding TaqMan probe (*mecA*-probe) for qPCR assay for *mecA* were also synthesized based on a previous report (Table 1) [26]. All the primers and probes designed in this study are summarized in Table 1 and were synthesized by BGI TechSolutions Co., Ltd., Beijing, China.

### 2.4. Real-Time RAA Assay

The real-time RAA assays were performed in a 25 μL reaction volume using an RAA exo kit (Amp-Future Biotech Co., Ltd., Weifang, Shandong, China) according to the manufacturer’s instructions. The reaction mixture included 14.7 μL of Buffer A, 1.25 μL of Buffer B, 1.0 μL of each primer pair (10 μM), 0.3 μL of exo probe (10 μM), 5.75 μL of nuclease-free water, and 1.0 μL of the nucleic acid template. Primer concentration and probe concentration were optimized. Primer pair was diluted to 3 µM, 4 µM, 5 µM, 6 µM, 7 µM, and 8 µM. Probe was diluted to 1 µM, 3 µM, 5 µM, 7 µM, 9 µM. All reagents except the DNA template were added into the reaction tubes containing a dried enzyme pellet provided by the kit. After adding 1 μL of the nucleic acid template to the tubes, the tubes were closed carefully, vortexed shortly, and centrifuged briefly. They were immediately placed in QuantStudio 6 Flex qPCR system (ThermoFisher, Waltham, MA, USA) and incubated at 39 °C for 20 min (1 cycle per half min). The fluorescence signal was monitored in real time. Samples that gave rise to an exponential amplification curve above the negative control (nuclease-free water) threshold within 20 min were judged as positive.

### 2.5. qPCR Assay

The *mecA*-based qPCR assay was carried out in a previous report [26] using the QuantStudio 6 Flex qPCR system (ThermoFisher, Waltham, MA, USA) with minor modifications. Briefly, the reaction system included 10 μL of 2× SµperFast Probe Mixtµre (KangWei Century Co., Ltd., Beijing, China), 0.4 μL of each *mecA* primer (10 μM), 0.4 μL of *mecA*-probe (10 μM), 8.4 μL of nuclease-free water, and 0.4 μL of the nucleic acid template. Thermal cycling conditions involved an initial 30 s incubation at 95 °C, followed by 40 cycles of 95 °C for 10 s and 58 °C for 20 s. Samples that produced a cycle threshold (Ct) value of <36 were considered positive.

### 2.6. Analytical Specificity

To evaluate the specificity of real-time RAA assay in detecting *mecA*, the genome templates prepared from some clinically relevant bacteria in dairy, including MRSA (ATCC43300), MSSA (ATCC29213), *E. faecalis* (ATCC29212), *S. agalactiae* (ATCC13813), *E. coli* (ATCC25922), *C. jejuni* (ATCC33560), and *S. enteritidis*, were detected by the real-time RAA assay.

### 2.7. Analytical Sensitivity

The copy number of pUC57-*mecA* plasmid contained in each microliter standard was calculated as previously described [56]. Through a 10-fold gradient dilution, the DNA concentrations of pUC57-*mecA* ranging from 10^6^ to 10^0^ copies/μL were prepared as templates for RAA assay, and 1 μL of the template was added into each reaction tube.

### 2.8. Sample Detection

Total DNA extracted from 67 bovine-derived *S. aureus* isolates was submitted to real-time RAA assay and qPCR assay, respectively. Statistical analyses and data plotting were performed using the GraphPad Prism software (version 7.0; La Jolla, CA, USA) to determine their level of agreement.

## 3. Results

### 3.1. Design of the mecA Real-Time RAA Primers and Probe

To obtain the best diagnostic target for *mecA*, 954 *mecA* sequences of MRSA currently available in the NCBI database were aligned by using the MAFFT Alignment program of the Geneious Prime software (Biomatters Ltd., Auckland, New Zealand) to identify the highly conserved regions. As shown in Figure 1A, a region of about 300 bp (980~1280 nt, GenBank No. NG_047938.1) within *mecA* exhibited high conservation among all the aligned *mecA* sequences. It was selected as the amplification candidate for real-time RAA assay. There is generally a restriction of ideal probe locations to sequences in which two thymines (T) can be found with fewer than about five intervening nucleotides, and an optimal fragment was finally selected for the design of the exo probe p1073–1120. The two qualified T residues (T1103, T1105) within this probe are highly conserved among the 954 strains (Figure 1B). Meanwhile, three forward (F1:F1041–1071, F2:F1025–1054, and F3:F989–1020) and three reverse (R1:R1121–1150, R2:R1165–1194, and R3:R1181–1210) primers that surround the probe p1073–1120 were designed and synthesized for a primer screening assay to screen the best performing primer pair for the RAA assay (Figure 2A) (Table 1). Specifically, nine primer pairs were used to perform real-time RAA reactions by combining three upstream primers with three downstream primers. The screening experiments showed that, compared with the other eight primer pairs, primer pair F1/R3 had the best amplification performance with the fastest speed and the highest fluorescence value (Figure 2b). Therefore, primer pair F1/R3 and probe p1073–1120 were determined as the optimal combination and applied to the subsequent real-time RAA analysis.

### 3.2. Reaction Optimization for Real-Time RAA Assays

For an optimal RAA reaction system, the concentration of the primer pair and probe were evaluated through a concentration grading experiment, respectively. The primer concentration grading experiment showed, among the 2 µM, 3 µM, 4 µM, 5 µM, 6 µM, and 7 µM pairs (F1/R3), the amplification curve of the 5 µM primer pair exhibited the strongest fluorescence signal and the shortest reaction time (Figure 2C), and thus was selected as the optimal reaction concentration of primer pair for subsequent RAA assay. Similarly, a series of diluted probes p1073–1120 (1 µM, 3 µM, 5 µM, 7 µM, 9 µM) were tested to select the best concentration of the probe. As shown in Figure 2D, although the fluorescence value of the 9 µM probe was the highest, the reaction time of the 5 µM probe was the fastest. Its amplification value was close to that of the 9 µM probe to better meet the principle of economic and rapid detection of MRSA, and the optimal RAA probe concentration was determined at 5 µM.

### 3.3. Analytical Sensitivity of the Real-Time RAA Assays

**Figure 3:**
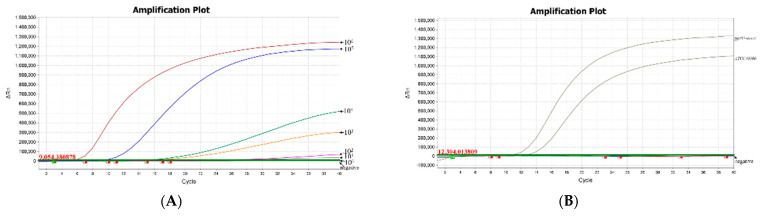
Sensitivity and specificity analysis of the RAA assay for *mecA.* (**A**) The analytical specificity of the RAA assay. Amplification curves in different colors are RAA reaction readouts to which serial dilutions of positive plasmids were added. The dilutions of positive plasmids were 10^6^ copies/reaction, 10^5^ copies/reaction, 10^4^ copies/reaction, 10^3^ copies/reaction, 10^2^ copies/reaction, 10^1^ copies/reaction, and 1 copy/reaction. (**B**) The analytical specificity of the RAA assay. Amplification curves in different colors are RAA reaction reads with the addition of genomic DNA templates from different bacteria, including MRSA (ATCC43300), MSSA, *E. faecalis*, *S. agalactiae*, *E. coli*, *C. jejuni*, *S.enteritidis*, the positive plasmid puc57-*mecA*, and negative control ddH_2_O.

We used 10-fold serially diluted positive plasmids as amplification templates to further evaluate the analytical sensitivity of the RAA assay. As shown in Figure 3A, the amplification results showed that the fluorescence value of the templates with different concentrations exhibited a dose-dependent effect within the above template concentration range. Compared with the negative control, a clear and reproducible amplification signal could still be detected when the positive plasmid concentration dropped to 10 copies/reaction. However, when the plasmid concentration reduced to one copy/reaction, the amplification signal disappeared; thus, the detection limit of real-time RAA was determined at 10 copies/reaction.

### 3.4. Analytical Specificity of the Real-Time RAA Assays

After completion of analytical sensitivity detection, analytical specificity was also performed by comparing the MRSA with other clinically relevant bacteria. As shown in Figure 3B, only nucleic acid samples from the MRSA reference strain (ATCC43300) and plasmid control were positive for the real-time RAA assays, while other bacteria tested such as *E. faecalis*, *S. agalactiae*, *E. coli*, *C. jejuni*, and *S. enteritidis* were negative. This result demonstrated that the real-time RAA assay we developed has reasonable analytical specificity for *mecA* detection.

### 3.5. Diagnostic Performance of Real-Time RAA Assays Evaluated with Clinical S. aureus Clinical Isolates

**Figure 4:**
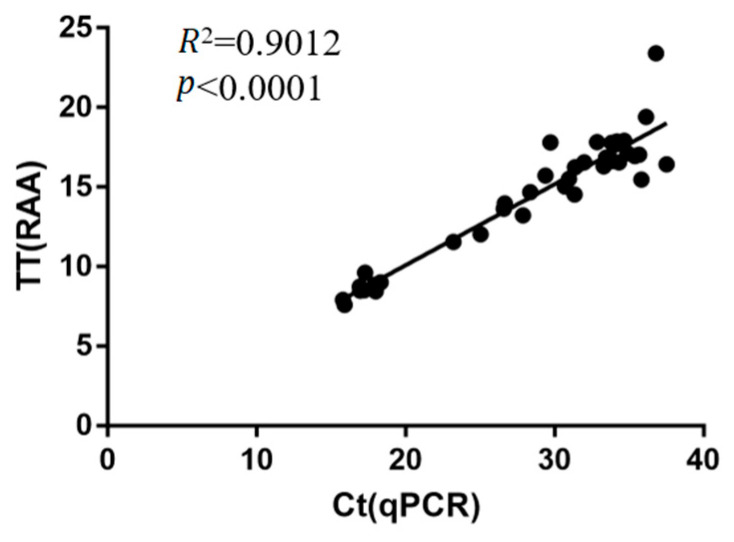
The correlation of real-time RAA and qPCR for MRSA diagnosis based on the detection results of 67 *S. aureus* clinical isolates. The threshold time (TT) values of real-time RAA at *Y*-axis and the cycle threshold (Ct) values of qPCR at *X*-axis were analyzed by linear regression analysis using GraphPad Prism software. The results from both assays were significantly correlated (*R*^2^ = 0.9012, *p* < 0.0001).

To further estimate the diagnostic performance of the real-time RAA assay in clinical practice, 67 clinical isolates of *S. aureus* from dairy farms clinically suspected of having *S. aureus* infection, which had been identified as *S. aureus* by a *nuc* PCR method [55] (Supplement Figure S1), were simultaneously detected by real-time RAA and TaqMan probe qPCR. The comparison results between the real-time RAA and qPCR assays are shown in Table 2. The data showed that 56 and 11 samples tested positive and negative for MRSA by RAA assay, respectively, while 54 and 13 samples tested positive and negative for MRSA by qPCR assay, respectively. Therefore, the overall agreement between qPCR and RAA in *S. aureus* clinical isolates detection was 97.01% (65/67). Further linear regression and Pearson correlation analyses revealed a significant correlation between qPCR and RAA, with an R^2^ value of 0.9012 (Figure 4), and the kappa value of the RAA assay was 0.9517 (*p* < 0.0001; Table 2).

## 4. Discussion

In this study, a real-time fluorescent RAA method for *mecA* was developed for the detection and screening of MRSA. The RAA method we established is mainly intended to be applied to detect MRSA in livestock farms or a rudimentary clinical laboratory. To ensure detection performance during the establishment of our real-time RAA assay, the most conserved region within the *mecA* gene was defined by multiple alignments of 954 strains of MRSA. Second, the specific primers and exo probe within the target region were manually designed by referring to the amplification guidelines proposed by kit instructions. After primer screening and concentration optimization of the primer and probe, the RAA assay’s analytical sensitivity and specificity were evaluated. To our satisfaction, the detection limit of this assay was tested to be 10 copies per reaction of positive plasmid, which is close to the detection limit of most qPCR methods and even higher than that of a LAMP-CRISPR assay for MRSA [57]. Additionally, the real-time RAA assay was specific for detecting MRSA and had few cross-reactivities to other clinically relevant bacteria on a dairy farm, such as *E. faecalis*, *S. agalactiae*, *E. coli*, *C. jejuni*, and *S. enteritidis*.

To further assess the diagnostic performance of the real-time RAA assay, 67 clinically isolated *S. aureus* strains from cows with mastitis were tested by the real-time RAA and a public TaqMan probe qPCR assay. Among clinical isolates of *S. aureus*, 56 and 54 isolates tested positive for MRSA by real-time RAA and qPCR, respectively. The overall agreement was 97.1% (65/67). Although two samples were positive in real-time RAA and negative in qPCR, the CT value of both samples was 37, indicating that these two samples may be weakly positive in the qPCR assay. When the genomes of these two samples obtained through the genome extraction kit were amplified by qPCR again, the result was positive (data not shown). The above experimental results showed that the real-time RAA for MRSA we established has good sensitivity and specificity matching with qPCR. In addition, this method also has the significant advantage of a short time. Generally, the real-time RAA assay can complete the detection within 20 min, while qPCR usually takes 1~2 h [18]. Undoubtedly, the prompt diagnosis of MRSA is essential for timely medical treatment and limiting the spread of MRSA. Another advantage of real-time RAA is the simplicity of operation. In addition, it can function efficiently over a wide temperature range, 37 °C to 42 °C, and can also be run using a simple temperature controller, such as water bath heating equipment. Therefore, our real-time RAA assay has the potential to be developed into a portable on-site kit for rapid MRSA detection or screening in rudimentary clinical laboratories or livestock farms.

## 5. Conclusions

In conclusion, a real-time fluorescent RAA assay targeting the conserved region within the *mecA* gene was developed and validated for MRSA detection. The developed assay has reasonable specificity and sensitivity consistent with qPCR, is faster and simpler to operate, and thus can provide a robust tool for the rapid detection of MRSA.

## Figures and Tables

**Table 1:** Sequences of primers and probes for MRSA real-time RAA and qPCR.

Name	Sequence (5′ 3′)	Gene	Location	Reference
*mecA*-F1	TACTGCTATCCACCCTCAAACAGGTGAATTA	*mecA*	1041–1071	This study
*mecA*-F2	ATGATTATGGCTCAGGTACTGCTATCCACC	*mecA*	1025–1054	This study
*mecA*-F3	CTAAAGTTCAAAAGAGTATTTATAACAACATG	*mecA*	989–1020	This study
*mecA*-R1	TTAATTTATTATATTCTTCGTTACTCATGC	*mecA*	1121–1150	This study
*mecA*-R2	TGTAATCTGGAACTTGTTGAGCAGAGGTTC	*mecA*	1165–1194	This study
*mecA*-R3	TTGAACCTGGTGAAGTTGTAATCTGGAACT	*mecA*	1181–1210	This study
p1073–1120	TAGCACTTGTAAGCACACCTTCATATGACG[FAM-dT] [thf][BHQ1-dT]ATCCATTTATGTATG	*mecA*	1073–1120	This study
*mecA*-F	GATTATGGCTCAGGTACTGCTATCC	*mecA*	1027–1051	[26]
*mecA*-R	TTCGTTACTCATGCCATACATAAATG	*mecA*	1109–1134	[26]
*mecA*-probe	VIC-CCTCAAACAGGTGAATTATTAGCACTTGTAAGCA-BHQ1	*mecA*	1053–1087	[26]

**Table 2:** Detection performance comparison between the *mecA*-based real-time RAA and qPCR assays for the detection of MRSA on *S. aureus* clinical isolates.

Method		qPCR			*Kappa*	*p*-Value
		Positive	Negative	Total		
RAA	Positive	54	2	56	0.9517	0.0001
	Negative	0	11	11		
Total		54	13	67		

## Data Availability

Data are available upon request.

## Supplementary Materials

The following supporting information can be downloaded at: https://www.mdpi.com/article/10.3390/microorganisms10122351/s1, Figure S1: PCR confirmation of *S. aureus* isolates showing amplification of the *nuc* gene.
