# Supplementary material for: Development of a Real-Time Recombinase-Aided Amplification Method to Rapidly Detect Methicillin-Resistant Staphylococcus aureus
Source: Microorganisms. 2022 Nov 28;10(12):2351. doi: 10.3390/microorganisms10122351 (PMC9784193; doi:10.3390/microorganisms10122351)
Supplement: Supplementary file 1 [file microorganisms-10-02351-s001.zip › microorganisms-1926329-supplementary.pdf]

Supporting information

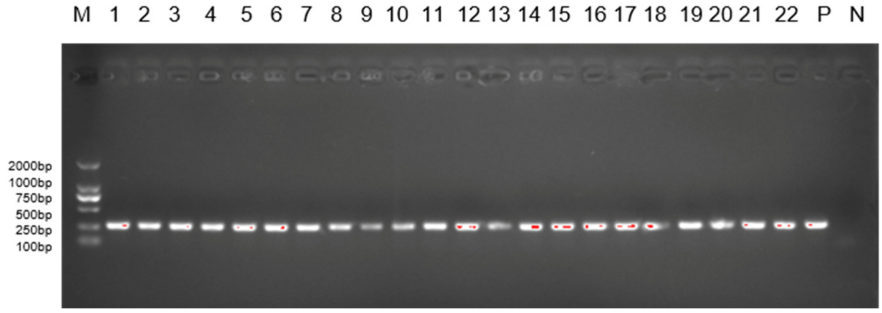

Figure S1. PCR confirmation of *S. aureus* isolates showing amplification of the *nuc* gene.
Lanes P, N and M represent positive control (ATCC29213), negative control (ATCC25922) and DNA marker DL2000 respectively, Lanes 1–22 show the *nuc* gene (270 bp) of *S. aureus* isolates 1–22.
